# Psychosocial risk factors for depression and anxiety in European postpartum women: A scoping review

**DOI:** 10.18332/ejm/216184

**Published:** 2026-01-24

**Authors:** Elīna Zelčāne, Anita Pipere, Kristīne Vende Kotova, Ance Mestere, Kristīne Mārtinsone

**Affiliations:** 1 Department of Health Psychology and Paedagogy, Rīga Stradiņš University, Riga, Latvia

**Keywords:** postpartum depression, psychosocial risk factors, Europe, postpartum anxiety

## Abstract

**INTRODUCTION:**

Postpartum depression (PPD) is common maternal mental health disorder, while postpartum anxiety (PPA) has recently increased. Comprehensive reviews on PPA psychosocial risk factors in Europe remain limited. This scoping review examines shared and distinct psychosocial risk factors for PPD and PPA among European postpartum women.

**METHODS:**

The review followed PRISMA guidelines, and Arksey and O’Malley’s scoping framework. ProQuest, Web of Science, Scopus, MEDLINE, Sage Journals were searched on 24 October 2025. Eligibility criteria were defined *a priori* using the Population–Concept–Context framework, and study selection was conducted using the Rayyan platform. Study quality was assessed using Mixed-Methods Appraisal, AMSTAR-2 tools, with relevance ensured through inclusion criteria and reviewer triangulation. An inductive, reflexive thematic analysis was applied to synthesize the identified factors.

**RESULTS:**

Following screening of 1219 records, 30 studies published between 2019 and 2024 were included. Of these, 21 examined PPD, three focused on PPA, and six addressed both conditions. Shared psychosocial risk factors included problematic partner relationships (n=13), insufficient support (n=9), and stressful life events (n=7). Risk factors more commonly associated with PPD were older maternal age (n=3), difficulties in emotion recognition (n=2), and isolation or loneliness (n=2). PPA-specific risk factors included lack of information and unpreparedness (n=2), unrealistic expectations (n=1), and internal or external stigma (n=1).

**CONCLUSIONS:**

Key psychosocial risk factors for PPD and PPA in Europe include problematic partner relationships, low support, stressful life events. Early identification and targeted interventions are crucial.

## INTRODUCTION

The postpartum period is a significant transition, marked by profound hormonal, physical, psychological and psychosocial changes, and is typically defined as extending up to one year after childbirth^1^. While many women adapt to these changes, not all can cope effectively due to a variety of risk factors that can lead to mental health disorders, such as postpartum depression (PPD) and postpartum anxiety (PPA)^1,2^. Unlike the transient ‘baby blues’, PPD is more severe, often manifesting as persistent sadness, low self-esteem, sleep disturbances, anxiety, and difficulties bonding with the baby^3^, and it can negatively affect both maternal and infant health, as well as the mother–infant relationship^4^. PPA is characterized by a heightened sense of worry or fear, which can manifest as symptoms of tension, fatigue, irritability, changes in concentration, and insomnia^5,6^.

The World Health Organization estimates that at least 13% of postpartum women develop a mental health disorder, with PPD being the most common^7^. Amer et al.^8^ suggest that the global prevalence of PPD may be even higher, potentially reaching 17%. Despite extensive research on PPD in the past decade, PPA is also becoming increasingly prevalent, especially after COVID-19, drawing growing attention from researchers and healthcare professionals^5^.

Reported prevalence rates of PPD and PPA differ widely across studies, mainly due to variations in diagnostic frameworks, measurement tools, and the timing of data collection. During the COVID-19 pandemic, globally, PPD and PPA increased by 1.54 and 2.56 times, respectively^9^. Meta-analyses suggest that the global pooled prevalence of PPD was approximately 26%, while PPA affected around 31% of women. Notably, Europe showed some of the highest prevalence rates of PPA, reaching up to 34%^9,10^. One explanation for the increase in PPA cases in Europe in recent years could be linked not only to the COVID-19 pandemic and the resulting distrust in the healthcare system but also to the war in Ukraine, which undermined the general sense of security.

The true prevalence of postpartum mental health disorders may be considerably higher, as research suggests that up to 50% of affected women remain undiagnosed and untreated^3^. Key reasons PPD is often underdiagnosed are stigma, limited access to care, and a lack of screening or awareness^11^. Similarly, PPA is frequently overlooked by both clinicians and patients because its symptoms overlap with typical postpartum experiences, making accurate identification more challenging^6^. With the rising prevalence of PPA, it is essential for postpartum research to explore risk factors for both PPD and PPA, as it has been proven that about 25% to 50% of women with anxiety disorders also show symptoms of PPD^12^.

This scoping review aims to address this gap by systematically examining the common risk factors shared by both conditions as well as distinct psychosocial risk factors for PPD and PPA separately among postpartum women in Europe. Although in health psychology, there is no singular approach that clearly defines what is included in psychosocial risk factors, they are generally understood as factors arising from the interaction between individuals and their social environment, including family, community, and broader societal structures^13^.

An extensive search of academic databases did not identify any European studies specifically examining psychosocial risk factors for both PPD and PPA. Since Europe has one of the highest prevalences of PPA during the COVID-19 pandemic and regional differences may influence mental health outcomes^14^, the decision was made to analyze risk factors specifically among women living in Europe. Despite cultural and healthcare differences, the European region shares unifying factors, such as the geopolitical context and common attitudes toward gender equality and family roles, which justify analyzing the European region as a whole in this scoping review^14^.

The research questions guiding this scoping review are: ‘What are the shared psychosocial risk factors for PPD and PPA among women living in the European region?’; ‘What are the psychosocial risk factors for PPD among women living in the European region?’; and ‘What are the psychosocial risk factors for PPA among women living in the European region?’.

## METHODS

### Design

A scoping review methodology was selected to outline the existing body of research and identify gaps that require further investigation. To address the research objective, this scoping review followed the five out of the six stages of the methodological framework outlined by Arksey and O’Malley^15^, which include: 1) identifying the research questions; 2) identifying relevant studies; 3) study selection; 4) charting the data; and 5) collating, summarizing, and reporting results.

This scoping review used the Preferred Reporting Items for Systematic Reviews and Meta-Analyses extension for Scoping Reviews (PRISMA-ScR) framework^16^. The framework was employed to structure the review process systematically, ensuring clarity, transparency, and consistency. The review aimed to map key concepts within the research area, identify existing knowledge gaps and provide a comprehensive overview of the literature on psychosocial risk factors associated with PPD and PPA. The PRISMA-ScR checklist can be found in Supplementary file Appendix 1. The scoping review protocol was developed *a priori* and registered on the Open Science Framework on 29 April 2025.

### Search strategy

Following Peters^17^, a three-phase search strategy was used to systematically explore the literature on psychosocial risk factors of PPD and PPA. In the first stage, a preliminary search was conducted in the ProQuest and MEDLINE databases to assess the literature volume and identify relevant search terms. The search strategy focused on key concepts such as ‘postpartum period’, ‘postpartum depression’, and ‘postpartum anxiety’. Keywords were combined using Boolean operators ‘OR’ and ‘AND’. The search employed four main search clusters (Table 1).

**Table 1:** Database search strategy and keywords used to identify studies on psychosocial risk factors for postpartum depression and postpartum anxiety among European postpartum women, 2019–2024

*Keywords*	*Search strings*
Postpartum period	‘postpartum period’ OR ‘postnatal period’
Postpartum depression	‘postpartum depression’ OR ‘postnatal depression’ OR ‘depression’
Postpartum anxiety	‘postpartum anxiety’ OR ‘postnatal anxiety’ OR ‘anxiety’
Psychosocial risk factors	‘psychosocial risk factor*’ OR ‘risk*’ OR ‘experience’

In the second stage, a search was conducted in evidence-based databases: ProQuest, Web of Science, Scopus, MEDLINE, and Sage Journals. The search targeted studies published in the last five years. The search strategy was carried out on 24 October 2024, and five years period was chosen to include studies both within and outside the COVID-19 pandemic period. In the third stage, the reference list of identified studies was systematically screened to assess which studies met the inclusion criteria for this scoping review.

### Eligibility criteria

The PCC (Population–Concept–Context) framework from the Joanna Briggs Institute^18^ was employed to define the search strategy, inclusion, and exclusion criteria. Eligible studies included European postpartum women (Population), examined psychosocial risk factors for PPD and/or PPA (Concept), and were conducted in European countries (Context). The primary criteria for database selection were that the studies: 1) were published between 1 January 2019 and 24 October 2024; 2) were written in English; 3) were conducted and published in Europe; and 4) were available in full-text format. Two independent reviewers judged article eligibility. The detailed inclusion and exclusion criteria are shown in Table 2.

**Table 2:** Inclusion and exclusion criteria applied to study selection in the scoping review of psychosocial risk factors for postpartum depression and postpartum anxiety among European postpartum women

	*Inclusion criteria*	*Exclusion criteria*
**Population**	Women in the postpartum period (up to 12 months postpartum) living in the European region (Albania, Andorra, Austria, Belarus, Belgium, Bosnia and Herzegovina, Bulgaria, Croatia, Cyprus, Czechia, Denmark, Estonia, Finland, France, Germany, Greece, Hungary, Iceland, Ireland, Italy, Kosovo, Latvia, Liechtenstein, Lithuania, Luxembourg, Malta, Moldova, Monaco, Montenegro, Netherlands, North Macedonia, Norway, Poland, Portugal, Romania, San Marino, Serbia, Slovakia, Slovenia, Spain, Sweden, Switzerland, United Kingdom)	Women in the prenatal period, men in the postpartum period, and women outside the European region were excluded. Russia, Turkey and Kazakhstan were excluded as they are only partially located in Europe, while Ukraine was excluded due to the ongoing military conflict, which could potentially impact the results.
**Diagnosis**	Diagnosis or positive screening for PPD or PPA	Symptoms of PPD or PPA which have neither been screened nor diagnosed
**Study outcome**	Studies focusing on the psychosocial risk factors for PPD or PPA	Studies addressing biological risk factors for PPD or PPA exclusively
**Study type**	Systematic review, literature review, scoping review, quantitative study, qualitative study, mixed-methods study	Study protocols, grey literature, studies that are not peer-reviewed
**Language**	Studies in English	Studies in other European languages

### Study selection

**Figure 1:**
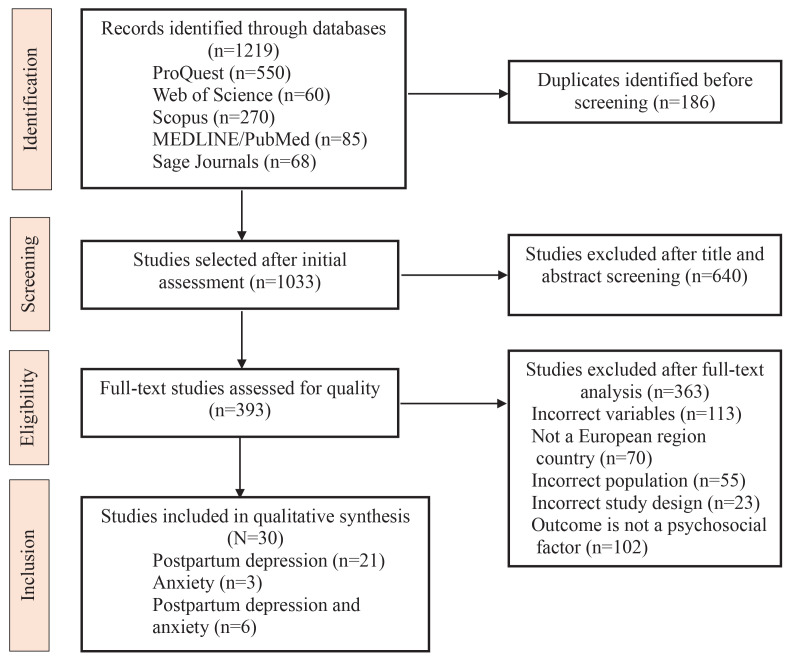
PRISMA-ScR flowchart of study selection for the scoping review of psychosocial risk factors for postpartum depression and postpartum anxiety in Europe, 2019–2024

The screening process was conducted in two stages. Initially, the titles and abstracts of relevant studies were reviewed, followed by a full-text assessment of the selected studies to determine eligibility. The subsequent data analysis included only studies that met all inclusion criteria. The Rayyan platform facilitated data management, including study organization, duplicate identification, and screening. Two researchers independently screened and selected the studies to ensure accuracy and consistency. Disagreements between the reviewers were resolved through discussions until a consensus was reached. The study selection process, including the number of studies initially identified, reasons for exclusion, and the final number of studies included in the review, is presented in the PRISMA-ScR flowchart (Figure 1).

### Quality appraisal

To enhance the transparency and interpretability of the findings, the methodological quality of the included studies was independently assessed by two reviewers. The methodological quality of the empirical studies was assessed using the Mixed-Methods Appraisal tool (2018)^19^, (Supplementary file Appendix 2). In addition, one of the included publications was an umbrella review, which was assessed using the AMSTAR-2 tool (2017)^20^, specifically developed for appraising systematic reviews and meta-analyses (Supplementary file Appendix 3).

### Data extraction and analysis

The Microsoft Excel spreadsheet was developed to determine the variables to be extracted from the reviewed articles. The following data were then extracted from each article: bibliographic information (first author, publication year), country, the title of the research, keywords, research objective, study design, assessment tools, type of disorder, study population and sample size, key findings and future research needs (Supplementary file Appendix 4). Data extraction was performed through triangulation, with two researchers independently extracting data to ensure accuracy and minimize bias. Any uncertainties were resolved through discussion, and when consensus could not be reached, a third researcher with the most extensive experience in qualitative research was consulted to ensure methodological rigor. The best options were then discussed and agreed upon collaboratively. Inductive thematic analysis, following the approach of Braun and Clarke^21^ (familiarization with the data, generating initial codes, searching for themes, reviewing themes, defining and naming themes, and writing the report), was applied to synthesize the extracted data. Using the triangulation between three researchers, the results were systematically grouped into categories derived from recurring themes, enabling the identification of both common and distinct psychosocial risk factors for PPD and PPA. This approach provided a structured overview of the key findings, highlighted the nuances within the data, and ensured the rigor and trustworthiness of the findings.

## RESULTS

### Description of included studies

The studies included in this scoping review comprised predominantly quantitative designs, mainly cross-sectional, with a few qualitative studies, as well as systematic reviews. Overall, the majority of empirical studies met key methodological quality criteria, such as clearly stated research objectives, appropriate study design, adequate reporting of sampling and data collection procedures, and the use of validated measurement tools.

The initial search yielded a total of 1219 titles. After identifying and eliminating 186 duplicate records, two reviewers screened the remaining 1033 abstracts. This process resulted in 393 articles being selected for full-text assessment. The primary exclusion criteria in this phase included irrelevance to the central concepts of the research, studies not originating from European countries, and inappropriate sample selection. Additionally, during the full-text screening phase, studies were excluded for the following reasons: outcomes not addressing psychosocial risk factors (n=102), inclusion of incorrect variables (n=113), studies conducted outside the European region (n=70), inappropriate study population (n=55), and unsuitable study design (n=23). Additional reasons for exclusion were insufficient methodological information, protocol-only publications, or lack of full-text availability. Thirty studies^22-51^ were deemed eligible and included in the scoping review, of which 21 focused solely on PPD, three on PPA, and six on both on PPD and PPA. Overall, the scoping review includes studies from 16 countries in the European region (Albania, Belgium, Cyprus, Denmark, Finland, France, Germany, Italy, Netherlands, Poland, Portugal, Serbia, Slovakia, Sweden, Switzerland, and the United Kingdom) published between 2019 and 2024 (Supplementary file Appendix 5).

**Figure 2:**
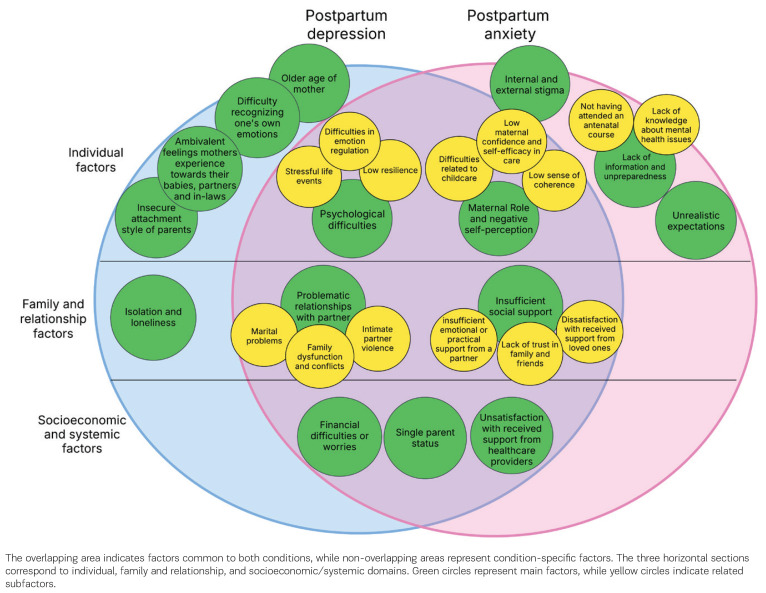
Conceptual overview of shared and condition-specific psychosocial risk factors for postpartum depression and postpartum anxiety among European postpartum women, 2019–2024

Studies encompassed a variety of psychosocial risk factors that are common for PPD and PPA among women in the European region. Figure 2 provides a graphical representation of both common and shared risk factors. The figure provides an integrated overview of all identified factors across domains, allowing for a comprehensive comparison between those associated with PPD and PPA.

### Shared psychosocial risk factors for PPD and PPA

Psychosocial risk factors common to both PPD and PPA were categorized into three main groups: individual factors, family and relationship factors, and socioeconomic and systemic factors.

*Individual factors*

Psychological difficulties are commonly mentioned as a group of individual risk factors for PPD and PPA. Among these, stressful life events (n=7; 23.33%) have been frequently reported. Specific stressors include unplanned pregnancy^13,22^, work-related stress^30,44^, and a negative childbirth experience, including complications such as prematurity^24,31,50^. Additionally, distress related to the child’s health^31,40^ and the perceived burden postpartum^22^ have been highlighted as contributing factors.

Difficulties in emotion regulation during pregnancy and postpartum^23,25,43^ (n=3; 10%) have also been associated with increased risk of PPD and PPA, as well as low resilience^37,48^ (n=2; 6.67%), which may influence a mother’s ability to cope with postpartum challenges.

In addition to psychological distress, challenges related to the maternal role and negative self-perception (n=5; 16.67%) have been linked to postpartum mental health difficulties. A low level of maternal self-efficacy in caregiving^32,36,48^ – defined as a mother’s belief in her ability to care for her infant and navigate the challenges of motherhood – as well as difficulties related to childcare^24,45,48^, such as frequent infant crying, maternal difficulty in understanding an infant’s cries^24,48^, and prenatal attachment difficulties^45^, have been reported as stressors contributing to PPD and PPA. Additionally, this group of risk factors includes a low sense of coherence^32^, which manifests as a reduced ability to perceive life events as comprehensible, manageable, and meaningful.

*Family and relationship factors*

Alongside individual factors, insufficient social support (n=9; 30%) is another frequently reported risk factor for PPD and PPA. In most studies, types of social support are not separately distinguished; however, some research identifies specific forms of support. It includes lack of emotional or practical support from a partner^24,28,29,31,41^, dissatisfaction with the support received from loved ones during pregnancy and childbirth^37^, and lack of trust in family and friends^28^.

Problematic family relationships (n=13; 43.33%) are also significant risk factors. In some studies, this factor was further categorized into subthemes such as marital problems^42,43,49^, intimate partner violence^34,43,46^ and family dysfunction and conflicts^32,42^.

*Socioeconomic and systematic factors*

Studies have shown that women experiencing financial difficulties or concerns about potential future financial challenges (n=6; 20%) are at a higher risk of developing PPD and PPA^24,28,37,42,43,50^. Financial insecurity can create additional stress, intensifying the emotional burden on new mothers.

Single-parent status^28,35,43,50^ (n=4; 13.33%) is another significant factor positively associated with the development of PPD^28,35,43,50^ and PPA^43^, as it often entails increased financial strain and the burden of managing daily responsibilities alone.

Additionally, dissatisfaction with the support received from healthcare providers during pregnancy, childbirth, and the postpartum period^36,37,47^ (n=3; 10%), has been identified as a contributing factor.

### Psychosocial risk factors for postpartum depression

In this scoping review, several risk factors were identified that are specific to depression but not anxiety. Among the individual factors, the following were mentioned: older age of the mother (n=3; 10%), which was identified in three studies^38,43,45^; difficulty recognizing one’s own emotions (n=2; 6.67%), identified in two studies^25,50^; ambivalent feelings mothers experience towards their babies, partners and in-laws (n=1) identified in one study^37^; and insecure attachment style of parents (n=1) identified in one study^23^.

Isolation and loneliness (n=2; 6.67%) were the only risk factors identified within the family and relationship factors domain, as highlighted in two studies^27,37^. Unlike the overlapping risk factors for both PPD and PPA – where insufficient support was commonly cited – the experience of depression was more often characterized by a complete absence of social support. No socioeconomic or systemic risk factors were identified as specific to PPD.

### Psychosocial risk factors for postpartum anxiety

The development of PPA is associated with several psychosocial risk factors that are not common for depression. In this study, only individual-level risk factors were identified. One such risk factor is lack of information and unpreparedness (n=2; 6.67%); this factor includes not having attended an antenatal course^28^, and lack of knowledge about mental health issues^36^. Another factor is internal and external stigma^36^, (n=1; 3.33%), which constitutes a significant barrier for new mothers, preventing them from seeking and receiving necessary support. In addition, unrealistic expectations (n=1; 3.33%) were identified as a risk factor specific to PPA. Although this risk factor was identified in only one study^36^, it warrants consideration as the only qualitative study in the scoping review, offering insight into women’s experiences. It includes expectations about childbirth and breastfeeding, unrealistic social comparisons, and social pressure. Unlike depression, where expectations reflect discrepancies in social support for childcare, anxiety-related expectations are linked to difficulties meeting perceived norms and standards reinforced by the idealized ‘perfect mother’ role.

## DISCUSSION

Addressing the first research question regarding the shared psychosocial risk factors for PPD and PPA among women living in the European region, the review found that there are far more shared risk factors for both mental health conditions than distinct ones. These shared risk factors include individual factors, like psychological difficulties, negative childbirth experiences, challenges in emotion regulation and low self-efficacy in the maternal role. From the family-related factors, the most common shared risk factors identified were problematic family relationships and insufficient social support. Additionally, socioeconomic challenges like financial insecurity and single-parent status were frequently mentioned as shared risk factors for both conditions.

One explanation for why so many risk factors are shared between PPD and PPA could be related to the fact that these two conditions are often comorbid, meaning they frequently occur together, or one follows the other as a response to similar hormonal changes as well as similar psychological and social stressors^52^. Significant life stressors, such as financial difficulties or relationship issues, are common precursors to both conditions^53^. These findings highlight the importance of multidisciplinary postpartum care models supporting individual, family and social domains.

Addressing the second research question about the psychosocial risk factors for PPD among women living in the European region, the findings revealed that, for new mothers experiencing depression, there is not only a difficulty in regulating emotions but also in recognizing them, as well as in managing conflicting emotional states. When emotions are not accurately recognized or understood, it becomes significantly more challenging to apply appropriate coping mechanisms, potentially leading to PPD^54^. These results underscore the potential benefit of mindfulness-based interventions or other structured programs, which may serve as a foundation for more effective emotion regulation in the postpartum period^55^.

Another risk factor for PPD is the older age of the mother. Although the underlying mechanisms of this increased risk are not yet fully understood, it has been proposed that older mothers may experience greater difficulties in adapting to the maternal role and may have limited access to peer support as their age diverges from socially normative expectations of motherhood^56^. To address this issue, age-inclusive support groups or peer-matching programs could provide tailored social and emotional resources for older mothers.

Among family-related factors, isolation and loneliness emerged as specific to PPD. To mitigate this problem, interventions could include implementing community-based support groups, psychiatric home visits, and strategies to increase partner involvement^57^.

Addressing the third research question about the psychosocial risk factors for PPA among women living in the European region, the findings revealed that in the case of PPA, women typically either lack information, which leads to uncertainty, or, conversely, have too much information, causing confusion, unrealistic expectations, and setting of standards upon themselves. Accurate and professional information during the postpartum period is crucial for ensuring women correctly understand their health and care needs^58^.

Comparing the studies in this scoping review is challenging, as some examined a range of risk factors while others focused on specific ones. Therefore, in the following discussion, we focused on analyzing the psychosocial risk factors for PPD and PPA across the European region as a whole while also offering insights into the types of factors reported in other parts of the world.

Overall, the results from the reviewed studies indicate that most of the psychosocial risk factors common for PPD and PPA have been previously discussed in other studies around the globe. No specific differences were observed in individual risk factors within the European region that had not also been identified in studies conducted in other parts of the world, such as in America or Asia.

In countries with developed economies, the psychosocial risk factors for PPD and PPA are generally similar; however, in developing countries, certain factors related to family and relationship dynamics, as well as socioeconomic and environmental influences, have been identified that were not reported in studies conducted in the European context.

Evaluating the individual factors of PPD and PPA identified in this scoping review with those conducted in various regions worldwide, reveals a generally consistent pattern. Findings indicate that psychological difficulties, like stressful life events^59^, negative childbirth experiences^60^ and emotion regulation difficulties^61^ are also frequently cited as common risk factors for both depression and anxiety in different regions of the world.

Examining the family and relationship factors reveals that a lack of social support is widely recognized as a common psychosocial risk factor, previously discussed in other systematic reviews and meta-analyses on PPD^59^ and PPA^62^. The findings of this scoping review are consistent with prior research in different world regions, highlighting the essential role of social support, especially from a partner during both pregnancy^63^ and the postpartum period^64^ in the prevention of PPD and PPA.

De Sousa Machado et al.^65^ analyzed data from research conducted across different regions of the world in their study on the role of social support for first-time mothers. They concluded that social support acts as a protective buffer against postpartum stressors, highlighting the importance of not only improving social support accessibility but also addressing the barriers that discourage women from seeking it. The authors’ conclusions are consistent with the findings of this scoping review, which also indicate that specific psychosocial barriers may inhibit women from seeking support. Specifically, two studies highlighted factors such as stigmatization, difficulties in normalizing the experience of postpartum disorders^37^, and challenges in recognizing emotions^25^, all of which may contribute to women’s hesitation to reach out for help.

In cases where a woman lacks sufficient support from her partner, she must have the opportunity to mobilize support within her social networks – such as other family members, friends, or the broader community – since instrumental support plays a significant role in meeting women’s basic needs during the postpartum period^66^. However, it should be noted that many families face the challenge of living far from their parents and relatives or do not maintain close enough relationships to seek help effectively.

Evagorou et al.^67^ have divided cultures into two groups: ethnokinship cultures (e.g. Eastern Asia, South Asia, Africa, Eastern Europe, and the Middle East) with strong family networks that provide primary maternal support, and technocentric cultures (e.g. USA, Canada, Western Europe, Great Britain, New Zealand, Australia). In the second group cultures, community-based support is less common, and women often leave the hospital without specific provisions. If necessary, women can seek support from mental health professionals. Most countries in this scoping review belong to technocentric cultures, where the nuclear family predominates, potentially limiting support. Therefore, special attention should be given to vulnerable women who live alone, face conflicts, and lack a strong social support network.

In light of these cultural differences and structural limitations, alternative sources of support have become increasingly important. According to one of the research studies included in this scoping review, the Internet can bridge the support gap by offering professional, evidence-based guidance and psychoeducation, helping to reduce the confusion and uncertainty experienced by women^36^. In addition to digital resources, more comprehensive antenatal preparation is needed to address the practical and psychological aspects of the perinatal period. Alongside practical preparations for childbirth, such as breathing exercises, labor techniques, and infant care classes, more focus should be placed on supporting women in addressing their emotional well-being^68^.

Focusing on socioeconomic and environmental factors, insufficient support from healthcare professionals is recognized as a risk factor for PPD and PPA globally and within Europe^69^. However, European women more often report concerns regarding the quality of received support^70^. In contrast, in developing countries, financial barriers to accessing medical care are a more prevalent risk factor^71^.

Slight regional variations were noted regarding financial difficulties as a risk factor for PPD and PPA. Studies conducted in regions with relatively high-income levels (Australia, Europe, North America) have shown that the risk for both PPD^72^ and PPA^62^ is higher in cases of low-income but financial difficulties are not among the dominant factors. Lack of social support and stressful life events are more common risk factors in high-income countries. Similarly, this scoping review identified financial difficulties as a risk factor (6 of 30 studies). However, they were not among the most prevalent factors. Conversely, in studies conducted in low-income countries outside Europe, financial difficulties are among the leading risk factors^73^.

This review also identified differences between the risk factors for PPD and PPA concerning emotional difficulties. PPD is often linked to feelings of hopelessness, social isolation and lack of emotional awareness, whereas PPA is characterized by excessive worry and unrealistic expectations. This difference is also supported by other studies not included in this scoping review, which mention that unrealistic expectations predict PPA symptoms^74^, while difficulties in emotional awareness predict PPD symptoms^75^.

This review highlights the crucial importance of the early identification of risk factors for mental health disorders. It is essential to train healthcare professionals, such as family physicians and pediatricians, in PPD screening, as research shows that healthcare providers have limited access to diagnostic techniques for identifying women at risk^76^. Moreover, by incorporating PPD screening in the ambulatory setting, screening rates increase, and providers gain confidence in discussing PPD with mothers^77^. Thus, the findings of this research have the potential to inform clinical practice by supporting early detection and management of postpartum mental health issues.

However, when considering postpartum mental health screening, we must not overlook the widespread prevalence of anxiety disorders. Anxiety is increasingly recognized not only as an independent risk factor but also as a comorbid condition alongside depression^12^. Given the significant overlap between PPA and PPD symptoms, it is essential that screening for postpartum mental health difficulties includes both conditions. The prevalence of PPA has notably increased in recent years, particularly following the COVID-19 pandemic^5^. However, the limited number of studies focusing exclusively on PPA suggests that research on this condition remains underdeveloped compared to PPD. This gap underscores the necessity for further investigations into the psychosocial determinants of PPA, particularly in the European context, where cultural and healthcare differences may influence maternal mental health outcomes.

### Strengths and limitations

The present review’s strengths include its focus on a previously unexplored research gap. An extensive search did not identify any previous scoping review specifically examining psychosocial risk factors for PPD and PPA within the European context. This regional focus provides valuable insights into the unique sociocultural and healthcare factors that may influence maternal mental health in Europe.

This review has several limitations. First, the inclusion was limited to articles published in English, potentially omitting relevant research in other European languages. Second, since many studies overlap with the COVID-19 pandemic, it is difficult to discern which psychosocial risk factors were explicitly influenced by pandemics and which represent enduring patterns. Third, only a small proportion of studies focused solely on PPA, limiting the review’s ability to draw firm conclusions about anxiety-specific risk factors. Fourth, a limitation of this review is the inability to determine causal relationships due to the high proportion of cross-sectional studies included. While these studies offer valuable insights into the associations, they do not permit conclusions regarding the directionality or causality of the identified risk factors. Finally, potential publication bias cannot be excluded, as grey literature and unpublished studies were not searched.

## CONCLUSIONS

Individual-level risks associated with PPD and PPA include stressful life events, emotional regulation difficulties, low resilience, and maternal self-doubt. Relational risks, particularly insufficient partner support, conflict, or violence, emerged as especially salient. Additionally, financial strain, single parenthood, and dissatisfaction with healthcare support further contributed to maternal distress.

Findings related to anxiety may not fully capture the distinct risk factors for PPA due to the small number of studies available. Nonetheless, distinct risk factors were identified for each condition. PPD-specific risks included older maternal age, difficulty identifying one’s own emotions, ambivalent feelings toward close others, and insecure attachment styles. Moreover, depression was more often associated with experiences of isolation and a complete absence of social support. In contrast, PPA-specific risks were limited to individual-level factors, including lack of information and preparedness, perceived stigma, and unrealistic expectations related to motherhood, particularly the pressure to meet idealized standards shaped by social and cultural norms.

The results obtained in this study may be used for clinical screening purposes and for the development of interventions targeting maternal mental health. The findings underscore the complex nature of postpartum mental health risks and emphasize the significance of social, psychological, and contextual factors. Future research should consistently disaggregate data on PPD and PPA and employ standardized reporting to improve comparability. Additional studies involving diverse populations and cultural contexts, particularly regarding PPA, are necessary to elucidate these risk factors and inform the development of targeted interventions.

## Supplementary Material



## Data Availability

The data supporting this research can be found within the article and in the Supplementary file.
