# Supplementary material for: Psychosocial risk factors for depression and anxiety in European postpartum women: A scoping review
Source: Eur J Midwifery. 2026 Jan 24;10:10.18332/ejm/216184. doi: 10.18332/ejm/216184 (PMC12831440; doi:10.18332/ejm/216184)
Supplement: Supplementary file 1 [file EJM-10-01-s1.pdf]

**Appendix 1. Preferred Reporting Items for Systematic reviews and Meta-Analyses extension for Scoping Reviews (PRISMA-ScR) Checklist**

| SECTION | ITEM | PRISMA-ScR CHECKLIST ITEM | REPORTED ON PAGE # |
|---|---|---|---|
| **TITLE** | | | |
| Title | 1 | Identify the report as a scoping review. | |
| **ABSTRACT** | | | |
| Structured summary | 2 | Provide a structured summary that includes (as applicable): background, objectives, eligibility criteria, sources of evidence, charting methods, results, and conclusions that relate to the review questions and objectives. | |
| **INTRODUCTION** | | | |
| Rationale | 3 | Describe the rationale for the review in the context of what is already known. Explain why the review questions/objectives lend themselves to a scoping review approach. | |
| Objectives | 4 | Provide an explicit statement of the questions and objectives being addressed with reference to their key elements (e.g., population or participants, concepts, and context) or other relevant key elements used to conceptualize the review questions and/or objectives. | |
| **METHODS** | | | |
| Protocol and registration | 5 | Indicate whether a review protocol exists; state if and where it can be accessed (e.g., a Web address); and if available, provide registration information, including the registration number. | |
| Eligibility criteria | 6 | Specify characteristics of the sources of evidence used as eligibility criteria (e.g., years considered, language, and publication status), and provide a rationale. | |
| Information sources* | 7 | Describe all information sources in the search (e.g., databases with dates of coverage and contact with authors to identify additional sources), as well as the date the most recent search was executed. | |
| Search | 8 | Present the full electronic search strategy for at least 1 database, including any limits used, such that it could be repeated. | |
| Selection of sources of evidence† | 9 | State the process for selecting sources of evidence (i.e., screening and eligibility) included in the scoping review. | |
| Data charting process‡ | 10 | Describe the methods of charting data from the included sources of evidence (e.g., calibrated forms or forms that have been tested by the team before their use, and whether data charting was done independently or in duplicate) and any processes for obtaining and confirming data from investigators. | |
| Data items | 11 | List and define all variables for which data were sought and any assumptions and simplifications made. | |
| Critical appraisal of individual sources of evidence§ | 12 | If done, provide a rationale for conducting a critical appraisal of included sources of evidence; describe the methods used and how this information was used in any data synthesis (if appropriate). | |
| Synthesis of results | 13 | Describe the methods of handling and summarizing the data that were charted. | |

| SECTION | ITEM | PRISMA-ScR CHECKLIST ITEM | REPORTED ON PAGE # |
|---|---|---|---|
| **RESULTS** | | | |
| Selection of sources of evidence | 14 | Give numbers of sources of evidence screened, assessed for eligibility, and included in the review, with reasons for exclusions at each stage, ideally using a flow diagram. | |
| Characteristics of sources of evidence | 15 | For each source of evidence, present characteristics for which data were charted and provide the citations. | |
| Critical appraisal within sources of evidence | 16 | If done, present data on critical appraisal of included sources of evidence (see item 12). | |
| Results of individual sources of evidence | 17 | For each included source of evidence, present the relevant data that were charted that relate to the review questions and objectives. | |
| Synthesis of results | 18 | Summarize and/or present the charting results as they relate to the review questions and objectives. | |
| **DISCUSSION** | | | |
| Summary of evidence | 19 | Summarize the main results (including an overview of concepts, themes, and types of evidence available), link to the review questions and objectives, and consider the relevance to key groups. | |
| Limitations | 20 | Discuss the limitations of the scoping review process. | |
| Conclusions | 21 | Provide a general interpretation of the results with respect to the review questions and objectives, as well as potential implications and/or next steps. | |
| **FUNDING** | | | |
| Funding | 22 | Describe sources of funding for the included sources of evidence, as well as sources of funding for the scoping review. Describe the role of the funders of the scoping review. | |

JBI = Joanna Briggs Institute; PRISMA-ScR = Preferred Reporting Items for Systematic reviews and Meta-Analyses extension for Scoping Reviews.

* Where *sources of evidence* (see second footnote) are compiled from, such as bibliographic databases, social media platforms, and Web sites.

† A more inclusive/heterogeneous term used to account for the different types of evidence or data sources (e.g., quantitative and/or qualitative research, expert opinion, and policy documents) that may be eligible in a scoping review as opposed to only studies. This is not to be confused with *information sources* (see first footnote).

‡ The frameworks by Arksey and O'Malley (6) and Levac and colleagues (7) and the JBI guidance (4, 5) refer to the process of data extraction in a scoping review as data charting.

§ The process of systematically examining research evidence to assess its validity, results, and relevance before using it to inform a decision. This term is used for items 12 and 19 instead of "risk of bias" (which is more applicable to systematic reviews of interventions) to include and acknowledge the various sources of evidence that may be used in a scoping review (e.g., quantitative and/or qualitative research, expert opinion, and policy document).

*From:* Tricco AC, Lillie E, Zarin W, O'Brien KK, Colquhoun H, Levac D, et al. PRISMA Extension for Scoping Reviews (PRISMA-ScR): Checklist and Explanation. Ann Intern Med. ;169:467–473. doi: 10.7326/M18-0850

**Appendix 2.** Methodological quality assessment criteria based on the Mixed Methods Appraisal Tool (MMAT, 2018) applied in the scoping review of psychosocial risk factors for postpartum depression and postpartum anxiety among European postpartum women

| | | All studies | | Qualitative studies | | | | | Quantitative Non-randomized | | | | | Quantitative descriptive | | | | | Mixed methods | | | | | |
|---|---|---|---|---|---|---|---|---|---|---|---|---|---|---|---|---|---|---|---|---|---|---|---|---|
| | First author, publication date | S1 | S2 | 1.1 | 1.2 | 1.3 | 1.4 | 1.5 | 3.1 | 3.2 | 3.3 | 3.4 | 3.5 | 4.1 | 4.2 | 4.3 | 4.4 | 4.5 | 5.1 | 5.2 | 5.3 | 5.4 | 5.5 | |
| 1. | Agnafors et al., 2023 | Y | Y | | | | | | Y | Y | Y/N | Y/N | Y | | | | | | | | | | | |
| 2. | Axfors et al., 2019 | Y | Y | | | | | | Y | Y | Y/N | Y/N | Y | | | | | | | | | | | |
| 3. | Balles et al., 2023 | Y | Y | | | | | | Y | Y | Y/N | Y | Y | | | | | | | | | | | |
| 4. | Brandao et al., 2024 | Y | Y | | | | | | Y | Y | N/A | Y/N | Y | | | | | | | | | | | |
| 5. | Brik et al., 2022 | Y | Y | | | | | | Y | Y | Y/N | Y | Y | | | | | | | | | | | |
| 6. | Caetano et al., 2022 | Y | Y | | | | | | | | | | | Y | Y | Y | N/A | Y | | | | | | |
| 7. | Camoni et al., 2022 | Y | Y | | | | | | Y/N | Y | N/A | Y | Y | | | | | | | | | | | |
| 8. | Cena et al., 2021 | Y | Y | | | | | | Y/N | Y | Y/N | Y | Y | | | | | | | | | | | |
| 9. | Clayborne et al., 2022 | Y | Y | | | | | | Y | Y | Y/N | Y | Y | | | | | | | | | | | |
| 10. | Doncarli et al., 2024 | Y | Y | | | | | | Y | Y | Y/N | Y | Y | | | | | | | | | | | |
| 11. | Feligreras-Alcala et al., 2020 | Y | Y | | | | | | | | | | | Y | Y/N | Y | N/A | Y | | | | | | |
| 12. | Fredriksen et al., 2019 | Y | Y | | | | | | Y | Y | Y/N | Y | Y | | | | | | | | | | | |

**Appendix 2.** Methodological quality assessment criteria based on the Mixed Methods Appraisal Tool (MMAT, 2018) applied in the scoping review of psychosocial risk factors for postpartum depression and postpartum anxiety among European postpartum women

| | | | | | | | | | | | | | | | | | | | | | | | | |
|---|---|---|---|---|---|---|---|---|---|---|---|---|---|---|---|---|---|---|---|---|---|---|---|---|
| 13. | Gastaldon et al., 2022 | | | | | | | | | | | | | | | | | | | | | | | |
| 14. | Hamimatunnisa et al., 2020 | Y | Y | | | | | | Y | Y | Y/N | Y | Y | | | | | | | | | | | |
| 15. | Harrisson et al., 2020 | Y | Y | Y | Y | Y | Y | Y | | | | | | | | | | | | | | | | |
| 16. | Holopainen & Hakulinen, 2019 | Y | Y | Y | Y | Y | Y/N | Y | | | | | | | | | | | | | | | | |
| 17. | Kazmierczak et al., 2020 | Y | Y | | | | | | Y/N | Y | Y/N | Y/N | Y | | | | | | | | | | | |
| 18. | Kiviruusu et al., 2020 | Y | Y | | | | | | Y/N | Y | Y/N | Y | Y | | | | | | | | | | | |
| 19. | Klein et al., 2023 | Y | Y | | | | | | Y | Y | Y/N | Y | Y | | | | | | | | | | | |
| 20. | Lelievre et al., 2021 | Y | Y | | | | | | Y/N | Y | Y/N | Y/N | Y | | | | | | | | | | | |
| 21. | Luciano et al., 2022 | Y | Y | | | | | | Y/N | Y | Y/N | Y | Y | | | | | | | | | | | |
| 22. | Jimenez-Barragan et al., 2024 | Y | Y | | | | | | Y | Y | Y/N | Y | Y | | | | | | | | | | | |
| 23. | Molgora et al., 2022 | Y | Y | | | | | | Y/N | Y | Y/N | Y | Y | | | | | | | | | | | |
| 24. | Smorti et al., 2019 | Y | Y | | | | | | Y/N | Y | Y/N | Y | Y | | | | | | | | | | | |
| 25. | Sunnqvist et al., 2019 | Y | Y | | | | | | Y | Y | Y/N | Y/N | Y | | | | | | | | | | | |

**Appendix 2.** Methodological quality assessment criteria based on the Mixed Methods Appraisal Tool (MMAT, 2018) applied in the scoping review of psychosocial risk factors for postpartum depression and postpartum anxiety among European postpartum women

| | | | | | | | | | | | | | | | | | | | | | | | | |
|---|---|---|---|---|---|---|---|---|---|---|---|---|---|---|---|---|---|---|---|---|---|---|---|---|
| 26. | Urbanova et al., 2021 | Y | Y | | | | | | Y | Y | Y/N | Y | Y | | | | | | | | | | | |
| 27. | Zee-van-den-berg et al., 2021 | Y | Y | | | | | | Y | Y | Y/N | Y | Y | | | | | | | | | | | |
| 28. | Zejnullahu et al., 2020 | Y | Y | | | | | | Y | Y | Y/N | Y | Y | | | | | | | | | | | |
| 29. | Zikic et al., 2024 | Y | Y | | | | | | Y/N | Y | Y/N | Y | Y | | | | | | | | | | | |
| 30. | Zyrek et al., 2024 | Y | Y | | | | | | Y | Y | Y/N | Y | Y | | | | | | | | | | | |

**Code Meaning**

**Y** - The criterion is fully met

**N** - The criterion is not met

**Y/N** - The criterion is partially met or met with limitations

**N/A** - Not enough information to assess the criterion

**S1**: Are there clear research questions? **S2:** Do the collected data allow to address the research questions? **1.1**: Is the qualitative approach appropriate to answer the research question? **1.2**: Are the qualitative data collection methods adequate to address the

**Appendix 2.** Methodological quality assessment criteria based on the Mixed Methods Appraisal Tool (MMAT, 2018) applied in the scoping review of psychosocial risk factors for postpartum depression and postpartum anxiety among European postpartum women

research question? **1.3**: Are the findings adequately derived from the data? **1.4**: Is the interpretation of results sufficiently substantiated by data? **1.5**: Is there coherence between qualitative data sources, collection, analysis and interpretation? **3.1**: Are the participants representative of the target population? **3.2**: Are measurements appropriate regarding both the outcome and intervention (or exposure)? **3.3**: Are there complete outcome data? **3.4**: Are the confounders accounted for in the design and analysis? **3.5**: During the study period, is the intervention administered (or exposure occurred) as intended? **4.1**: Is the sampling strategy relevant to address the research question? **4.2**: Is the sample representative of the target population? **4.3**: Are the measurements appropriate? **4.4**: Is the risk of nonresponse bias low? **4.5**: Is the statistical analysis appropriate to answer the research question? **5.1:** Is there an adequate rationale for using a mixed methods design to address the research question? **5.2**: Are the different components of the study effectively integrated to answer the research question? **5.3**: Are the outputs of the integration of qualitative and quantitative components adequately interpreted? **5.4**: Are divergences and inconsistencies between quantitative and qualitative results adequately addressed? **5.5**: Do the different components of the study adhere to the quality criteria of each tradition of the methods involved?

Appendix 3. Methodological quality assessment criteria based on the AMSTAR-2 tool applied to systematic reviews included in the scoping review of psychosocial risk factors for postpartum depression and postpartum anxiety among European postpartum women

**1. Did the research questions and inclusion criteria for the review include the components of PICO?**

| For Yes: | Optional (recommended) | |
|---|---|---|
| ☐ Population<br>☐ Intervention<br>☐ Comparator group<br>☐ Outcome | ☐ Timeframe for follow-up | ✓ Yes<br>☐ No |

**2. Did the report of the review contain an explicit statement that the review methods were established prior to the conduct of the review and did the report justify any significant deviations from the protocol?**

| For Partial Yes: | For Yes: | |
|---|---|---|
| The authors state that they had a written protocol or guide that included ALL the following:<br>☐ review question(s)<br>☐ a search strategy<br>☐ inclusion/exclusion criteria<br>☐ a risk of bias assessment | As for partial yes, plus the protocol should be registered and should also have specified:<br>☐ a meta-analysis/synthesis plan, if appropriate, *and*<br>☐ a plan for investigating causes of heterogeneity<br>☐ justification for any deviations from the protocol | ✓ Yes<br>☐ Partial Yes<br>☐ No |

**3. Did the review authors explain their selection of the study designs for inclusion in the review?**

| For Yes, the review should satisfy ONE of the following: | |
|---|---|
| ☐ *Explanation for* including only RCTs<br>☐ OR *Explanation for* including only NRSI<br>☐ OR *Explanation for* including both RCTs and NRSI | ✓ Yes<br>☐ No |

**4. Did the review authors use a comprehensive literature search strategy?**

| For Partial Yes (all the following): | For Yes, should also have (all the following): | |
|---|---|---|
| ☐ searched at least 2 databases (relevant to research question)<br>☐ provided key word and/or search strategy<br>☐ justified publication restrictions (e.g. language) | ☐ searched the reference lists / bibliographies of included studies<br>☐ searched trial/study registries<br>☐ included/consulted content experts in the field<br>☐ where relevant, searched for grey literature<br>☐ conducted search within 24 months of completion of the review | ☐ Yes<br>✓ Partial Yes<br>☐ No |

**5. Did the review authors perform study selection in duplicate?**

| For Yes, either ONE of the following: | |
|---|---|
| ☐ at least two reviewers independently agreed on selection of eligible studies and achieved consensus on which studies to include<br>☐ OR two reviewers selected a sample of eligible studies and achieved good agreement (at least 80 percent), with the remainder selected by one reviewer. | ✓ Yes<br>☐ No |

### 6. Did the review authors perform data extraction in duplicate?

For Yes, either ONE of the following:

- ☐ at least two reviewers achieved consensus on which data to extract from included studies
- ☐ OR two reviewers extracted data from a sample of eligible studies and achieved good agreement (at least 80 percent), with the remainder extracted by one reviewer.

☑ Yes
☐ No

### 7. Did the review authors provide a list of excluded studies and justify the exclusions?

For Partial Yes:

- ☐ provided a list of all potentially relevant studies that were read in full-text form but excluded from the review

For Yes, must also have:

- ☐ Justified the exclusion from the review of each potentially relevant study

☑ Yes
☐ Partial Yes
☐ No

### 8. Did the review authors describe the included studies in adequate detail?

For Partial Yes (ALL the following):

- ☐ described populations
- ☐ described interventions
- ☐ described comparators
- ☐ described outcomes
- ☐ described research designs

For Yes, should also have ALL the following:

- ☐ described population in detail
- ☐ described intervention in detail (including doses where relevant)
- ☐ described comparator in detail (including doses where relevant)
- ☐ described study's setting
- ☐ timeframe for follow-up

☑ Yes
☐ Partial Yes
☐ No

### 9. Did the review authors use a satisfactory technique for assessing the risk of bias (RoB) in individual studies that were included in the review?

**RCTs**

For Partial Yes, must have assessed RoB from

- ☐ unconcealed allocation, *and*
- ☐ lack of blinding of patients and assessors when assessing outcomes (unnecessary for objective outcomes such as all-cause mortality)

For Yes, must also have assessed RoB from:

- ☐ allocation sequence that was not truly random, *and*
- ☐ selection of the reported result from among multiple measurements or analyses of a specified outcome

☑ Yes
☐ Partial Yes
☐ No
☐ Includes only NRSI

**NRSI**

For Partial Yes, must have assessed RoB:

- ☐ from confounding, *and*
- ☐ from selection bias

For Yes, must also have assessed RoB:

- ☐ methods used to ascertain exposures and outcomes, *and*
- ☐ selection of the reported result from among multiple measurements or analyses of a specified outcome

☑ Yes
☐ Partial Yes
☐ No
☐ Includes only RCTs

### 10. Did the review authors report on the sources of funding for the studies included in the review?

For Yes

- ☐ Must have reported on the sources of funding for individual studies included in the review. Note: Reporting that the reviewers looked for this information but it was not reported by study authors also qualifies

☑ Yes
☐ No

**11. If meta-analysis was performed did the review authors use appropriate methods for statistical combination of results?**

**RCTs**
For Yes:

- ☐ The authors justified combining the data in a meta-analysis
  - ☐ AND they used an appropriate weighted technique to combine study results and adjusted for heterogeneity if present.
  - ☐ AND investigated the causes of any heterogeneity

☐ Yes
☐ No
☐ No meta-analysis conducted

**For NRSI**
For Yes:

- ☐ The authors justified combining the data in a meta-analysis
  - ☐ AND they used an appropriate weighted technique to combine study results, adjusting for heterogeneity if present
  - ☐ AND they statistically combined effect estimates from NRSI that were adjusted for confounding, rather than combining raw data, or justified combining raw data when adjusted effect estimates were not available
  - ☐ AND they reported separate summary estimates for RCTs and NRSI separately when both were included in the review

[x] Yes
☐ No
☐ No meta-analysis conducted

**12. If meta-analysis was performed, did the review authors assess the potential impact of RoB in individual studies on the results of the meta-analysis or other evidence synthesis?**

For Yes:

- ☐ included only low risk of bias RCTs
- ☐ OR, if the pooled estimate was based on RCTs and/or NRSI at variable RoB, the authors performed analyses to investigate possible impact of RoB on summary estimates of effect.

☐ Yes
[x] No
☐ No meta-analysis conducted

**13. Did the review authors account for RoB in individual studies when interpreting/ discussing the results of the review?**

For Yes:

- ☐ included only low risk of bias RCTs
- ☐ OR, if RCTs with moderate or high RoB, or NRSI were included the review provided a discussion of the likely impact of RoB on the results

☐ Yes
[x] No

**14. Did the review authors provide a satisfactory explanation for, and discussion of, any heterogeneity observed in the results of the review?**

For Yes:

- ☐ There was no significant heterogeneity in the results
- ☐ OR if heterogeneity was present the authors performed an investigation of sources of any heterogeneity in the results and discussed the impact of this on the results of the review

[x] Yes
☐ No

**15. If they performed quantitative synthesis did the review authors carry out an adequate investigation of publication bias (small study bias) and discuss its likely impact on the results of the review?**

For Yes:

- ☐ performed graphical or statistical tests for publication bias and discussed the likelihood and magnitude of impact of publication bias

[x] Yes
☐ No
☐ No meta-analysis conducted

**16. Did the review authors report any potential sources of conflict of interest, including any funding they received for conducting the review?**

| For Yes: | |
|---|---|
| ☐ The authors reported no competing interests OR | ☑ Yes |
| ☐ The authors described their funding sources and how they managed potential conflicts of interest | ☐ No |

**To cite this tool:** Shea BJ, Reeves BC, Wells G, Thuku M, Hamel C, Moran J, Moher D, Tugwell P, Welch V, Kristjansson E, Henry DA. AMSTAR 2: a critical appraisal tool for systematic reviews that include randomised or non-randomised studies of healthcare interventions, or both. BMJ. 2017 Sep 21;358:j4008.

Appendix 4. Data extraction framework and variables charted from the included studies in the scoping review of psychosocial risk factors for postpartum depression and postpartum anxiety among European postpartum women

| Study | Reference | Country | Title | Study focus | Research method | Types of articles | Measures | Study population | Aim/ purpose | Findings | Conclusion |
|---|---|---|---|---|---|---|---|---|---|---|---|
| 1 | Agnafors et al., 2023 | Sweden | Factors associated with maternal depressive symptoms up to 25 years after childbirth: A longitudinal prospective cohort study | Depression | Quantitative research | Journal articles/original research | Edinburgh Postnatal Depression Scale (EPDS), Hopkins Symptoms Checklist (HSCL-25), The Patient Health Questionnaire-9 (PHQ-9). | A total of 450 mothers participated in all three follow-ups (3 months postpartum, 12 year and 25 year follow-up). | The aim of the study was to identify factors associated with repeated self-reports of depressive symptoms. The hypothesis was that factors such as young age at childbirth, divorce, unemployment, low social support, prior depression, or experience of stressful life events would increase the risk of repeatedly reporting depressive symptoms. | High life stress around pregnancy and childbirth and earlier depressive symptoms were associated with depressive symptoms at the 25-year follow-up. Similarly, high life stress around pregnancy and childbirth and the experience of divorce were associated with repeated self-reports of depressive symptoms. | Stressful life events are important predictors of depressive symptoms and repeated episodes of depressive symptoms from the postnatal period until 25 years later. Moreover, previous depressive symptoms increase the risk for subsequent episodes. The results further stress the importance of identifying mothers experiencing depressive symptoms postpartum to enable treatment and early intervention. |
| 2 | Axfors et al., 2019 | Sweden | Cohort profile: the Biology, Affect, Stress, Imaging and Cognition (BASIC) study on perinatal depression in a population-based Swedish cohort | Depression | Quantitative research | Journal articles/original research | Edinburgh Postnatal Depression Scale (EPDS) | 5492 women are followed-up with data collection points around gestational week 32, at childbirth, as well as three times postpartum: after 6weeks, 6months and 1 year. | The study aims to investigate the biopsychosocial aetiological processes involved in perinatal depression (PND) and to pinpoint its predictors in order to improve early detection. | Neuroticism and attachment anxiety have been identified as important, and not entirely overlapping, predisposing psychological factors for postpartum depressive symptoms. | These adjusted associations in a prospective material provide support for a model of personality as a vulnerability factor for PND and suggest benefits of combining perspectives from the largely separated fields of hormonal stress responses, personality and attachment in the study of PND aetiology. |
| 3 | Bales et al., 2023 | France | Pathways between Risk/Protective Factors and Maternal Postnatal Depressive Symptoms: The ELFE Cohort | Depression | Quantitative research | Journal articles/original research | Edinburgh Postnatal Depression Scale (EPDS) | 11,583 mothers at 2 months postpartum | The present study aimed to explore the pathways between a wide range of risk and protective maternal, infant and environmental factors present both before and after birth and the severity of PNDS at 2 months postpartum in a | A lack of a partner's perceived antenatal emotional support, consultation with a mental health specialist before pregnancy, family financial difficulties, prenatal psychological distress and a difficult pregnancy experience were directly associated with the severity of maternal PNDS at 2 months PP, as well as lack of perceived postnatal support. | Regarding infant and parenthood characteristics, infant self-regulation difficulties, maternal difficulty in understanding infant crying and infant hospitalisation were directly associated with PNDS severity at 2 months PP, while maternal difficulty in understanding an infant's cries was also indirectly associated with infant self-regulation difficulties. |

Appendix 4. Data extraction framework and variables charted from the included studies in the scoping review of psychosocial risk factors for postpartum depression and postpartum anxiety among European postpartum women

| | | | | | | | | | | | |
|---|---|---|---|---|---|---|---|---|---|---|---|
| | | | | | | | | | large sample of women from the general population. | | |
| 4 | Brandao et al., 2024 | Portugal | Social Support and Postpartum Depressive Symptoms in Portuguese Women: The Mediating Role of Emotion Regulation Difficulties | Depression | Quantitative research | Journal articles/original research | Multidimensional Scale of Perceived Social Support (MSPSS), the Difficulties in Emotion Regulation Scale—Short Form (DERS-SF), Edinburgh Postnatal Depression Scale (EPDS). | 160 women participated in the study. Four times postpartum: 0–3 months; 4–6 months; 7–9 months; 10–12 months. | This study aimed to investigate the relationship between social support and postpartum depression symptoms, with a focus on the mediating role of emotion regulation difficulties. | The results indicated that lower levels of social support were significantly associated with greater difficulties in emotion regulation. Emotion regulation difficulties were significantly associated with more postpartum depressive symptom. Perceived social support was significantly associated with fewer postpartum depressive symptoms and fewer emotion regulation difficulties. Fewer emotion regulation difficulties were significantly associated with fewer postpartum depressive symptoms. | These findings underscore the critical role of both social support and emotion regulation in the development of PPD symptoms. Enhancing emotion regulation skills, particularly for women with limited social support, could be a key target for interventions aimed at reducing the risk and severity of PPD. |
| 5 | Brik et al., 2022 | Spain | Social Support and Mental Health in the Postpartum Period in Times of SARS-CoV-2 Pandemic: Spanish Multicentre Cohort Study | Anxiety and depression | Quantitative research | Journal articles/original research | Edinburgh Postnatal Depression Scale (EPDS), the State-Trait Anxiety Inventory (STAI) Scale, the Medical Outcomes Study Social Support Survey (MOS-SSS), and the Postpartum Bonding Questionnaire (PBQ). | 428 women during the first month after childbirth. | To explore the depression and anxiety symptoms in the postpartum period during the SARS-CoV-2 pandemic and to identify potential risk factors. | A lower level of social support (MOS-SSS), a fetal malformation diagnosis and a history of depression were independent risk factors for postpartum depression. A lower level of social support and a history of mental health disorders were independent risk factors for postpartum anxiety. | Finally, a low level of social support leads to increased levels of both anxiety and depression symptoms during the postpartum period, and to an increased risk of mother-to-infant bonding disorder. To conclude, the SARS-CoV-2 pandemic might have an impact on the risk of depression and anxiety symptoms during the postpartum period. In addition, women with low levels of social support are at a higher risk of both depression and anxiety during this period. |
| 6 | Caetano et al., 2022 | Portugal | Mattering and Depressive Symptoms in Portuguese Postpartum Women: The Indirect Effect of Loneliness | Depression | Quantitative research | Journal articles/original research | Sociodemographic, Clinical, and Infant's Characteristics, Edinburgh Postnatal Depression Scale (EPDS), Loneliness Scale (ULS-6), General Mattering Scale | 530 postpartum women being a mother in the last 12 months. | The current study aimed to understand the relationship between mattering, loneliness and depressive symptoms in Portuguese postpartum women and to examine the potential mediating role of loneliness in the relationship between mattering and depressive | It was found that the relationships between mattering, loneliness, and depressive symptoms were significant: (a) higher levels of mattering were associated with lower levels of loneliness and depressive symptomatology and (b) higher levels of loneliness were associated with higher levels of depressive symptomatology. It was found that mattering was significantly and negatively associated with loneliness and with depressive symptomatology. | These results highlight the importance of studying loneliness as a possible risk factor for postpartum depression and alert to the pertinence of considering mattering and loneliness in the assessment and intervention with women in the perinatal period. |

| | | | | | | | | | | | |
|---|---|---|---|---|---|---|---|---|---|---|---|
| | | | | | | | (GMS; Psychometric Studies of Portuguese Version Ongoing). | | symptomatology among postpartum women. | | |
| 7 | Cameroni et al., 2022 | Italy | The Impact of the COVID-19 Pandemic on Women's Perinatal Mental Health: Preliminary Data on the Risk of Perinatal Depression/Anxiety from a National Survey in Italy | Anxiety and depression | Quantitative research | Journal articles/original research | General Anxiety Disorder-7 (GAD-7), Edinburgh Postnatal Depression Scale (EPDS). | 12,479 women women during pregnancy and up to 12 months in the postpartum period. | The aim of the study is to estimate the prevalence of risk for both maternal depression and anxiety among women attending 18 healthcare centres in Italy during the SARS-COV-2 pandemic and to investigate the psychosocial risks and protective factors associated. | The variables found to be significant with depression were as follows: having economic problems, living alone, not being able to rely on support from relatives or friends, and not being able to rely on support from a partner. Those associated with the risk of anxiety include being Italian, having an education below secondary school level, having some or many economic problems, being unable to rely on support from relatives or friends, and not having attended an antenatal course. All three variables included in both models were significant for depression and anxiety, respectively, women currently taking psychotropic drugs, women with a past diagnosis of depression or anxiety, and women with family members diagnosed with depression or anxiety in the past. | The data from this survey could be useful to determine the impact of the SARS-COV-2 pandemic on women and to establish a screening program with common and uniformly applied criteria which are consistent with national and international women's mental health programs. |
| 8 | Cena et al., 2021 | Italy | Prevalence of Maternal Postnatal Anxiety and Its Association With Demographic and Socioeconomic Factors: A Multicentre Study in Italy | Anxiety | Quantitative research | Journal articles/original research | Psychosocial and Clinical Assessment Form (also covering demographic and socioeconomic factors), State-Trait Anxiety Inventory. | The assessment included 307 mothers at 1–24 weeks postpartum, and at >24 weeks postpartum. | This study (a) assesses the prevalence of maternal postnatal anxious symptomatology, and (b) analyses its association with demographic and socioeconomic variables as well as obstetric and other psychosocial variables. | Results showed a significantly higher risk of anxiety in mothers who had depression or anxiety during pregnancy, lack or enough psychological support from the partner, and high educational level (university degree or above). | As regards the other variables, the findings indicated antenatal depression or anxiety, parity, and current psychological support from the partner as having the strongest relationships. Nevertheless, this article reveals important findings. In particular, high anxious symptoms are common among new mothers, significantly more common than depressive symptoms. The results of this study will serve as a baseline for future comparisons between Western countries, as well as for the abovementioned future research on the prevalence/incidence of postnatal anxiety and its protective and risk factors, especially during the current COVID-19 pandemic period. |
| 9 | Clayborne et al., 2022 | Norway | Prenatal work stress is associated with prenatal and postnatal depression | Anxiety and depression | Quantitative research | Journal articles/original research | the Hopkins Symptom Checklist-25 (SCL-25), Edinburgh Postnatal | 77,999 women 76 months postpartum. | The objective of this study was to examine the associations between prenatal work stress and subsequent depression and anxiety. | At 30 weeks gestation and 6 months postpartum, prenatal work stress was positively associated with depression and anxiety after adjustment for baseline depression and anxiety, demographic and life- style factors, social support, and | Women dealing with work stress during pregnancy are more likely to experience subsequent depression and anxiety. Findings can inform the development of workplace strategies to support the mental health of expecting and new mothers. s a result, |

| | | | | | | | | | | | |
|---|---|---|---|---|---|---|---|---|---|---|---|
| | | | and anxiety: Findings from the Norwegian Mother, Father and Child Cohort Study (MoBa) | | | | Depression Scale (EPDS), the Symptom Checklist-8. | | | adverse events. Higher prenatal work stress was also associated with higher odds of moderate-severe depression at six months postpartum after covariate adjustment. Higher physical work stress was associated with increased depression and anxiety. | targeting work stress early in pregnancy may minimize risk towards future stress and depression. Employers, partners, and other available social supports can help pregnant women and new mothers mitigate the impacts of work- related stress by promoting supportive workplace cultures, balancing unpaid work between family members, and alleviating other potential sources of stress. |
| 10 | Doncarli et al., 2024 | France | Prevalence of anxiety symptoms and associated factors at 2 months postpartum, results from a 2021 French national prospective cohort study | Anxiety | Quantitative research | Journal articles/original research | Health Literacy Questionnaire (HLQ), Edinburgh Postnatal Depression Scale (EPDS). | 7,133 women 2 months postpartum | This study aimed to estimate PPA symptom prevalence at 2 months and to identify associated risk factors in a representative sample of all women who gave birth in France in 2021, and in two subgroups: women with no postpartum depression (PPD) symptoms, and those with no history of mental health care. | Two sociodemographic characteristics were associated with having PPA symptoms: being under 34 years of age and having lower health literacy. Medical or mental health care history included having a history of medical termination of pregnancy or psychiatric care since adolescence. | Estimated PPA symptom prevalence at 2 months in our study sample was 27.6%. The risk factors we identified may guide future prevention policies. |
| 11 | Feligreras-Alcala et al., 2020 | Spain | Personal and Family Resources Related to Depressive and Anxiety Symptoms in Women during Puerperium | Anxiety and depression | Quantitative research | Journal articles/original research | Edinburgh Postnatal Depression Scale (EPDS), STAI state anxiety questionnaire, Duke-UNC-11 (perceived social support), family APGAR (family funcioning), self-efficacy in care (Lawton), sense of coherence (SOC-13), perceived burden (Caregiver Strain Index) and stressful life events | This study includes 212 women at 6 weeks after delivery | objectives: (1) to determine point prevalence of depressive symptoms and anxiety in women at 6 weeks after delivery; (2) to establish the possible relationship between personal and family resources (i.e., perceived social support, family functioning, self-efficacy in care, sense of coherence and perceived burden) and depressive symptoms and anxiety in women in puerperium; and (3) to identify a possible risk profile for the development of postpartum depression and anxiety in women during puerperium. | A positive association was found between the presence of depressive symptoms and perceived burden and stressful life events. In addition, a negative association was found between depressive symptoms and family functioning, social support, sense of coherence and self-efficacy in care. In the regression model of depressive symptoms, independent variables were included together with the control variable, stressful life events. In this model, the presence of depressive symptoms is positively associated with the perceived burden and negatively with self-efficacy in care, social support and sense of coherence. | (1) depressive and anxiety symptoms in the puerperium period may be more prevalent than in other periods of a woman's life; (2) perceived social support, self-efficacy in caring for the newborn and sense of coherence can be protective factors for depressive symptoms and anxiety in the puerperium; (3) perceived burden in caring for the newborn may be a risk factor for the mentioned symptoms. In this study, the authors found that less perceived social support, greater perceived burden, less self-efficacy in care and a lesser sense of coherence are accompanied by increased depressive symptoms and anxiety in women in the puerperium (i.e., at 6 weeks after delivery), regardless of the presence of other stressful life events unrelated to the care of the newborn. |

Appendix 4. Data extraction framework and variables charted from the included studies in the scoping review of psychosocial risk factors for postpartum depression and postpartum anxiety among European postpartum women

| | | | | | | | | | | | |
|---|---|---|---|---|---|---|---|---|---|---|---|
| | | | | | | | (Holmes and Rahe). | | | | |
| 12 | Fredriksen et al., 2019 | Norway | Depressive Symptom Contagion in the Transition to Parenthood: Interparental Processes and the Role of Partner-Related Attachment | Depression | Quantitative research | Journal articles/Brief report | Edinburgh Postnatal Depression Scale (EPDS). | Data were collected from 1,036 mothers from midpregnancy until 12 months postpartum. | we aim to identify parents who are particularly vulnerable to the development of disruptive processes of negative mood states. we aim to provide a sound statistical approach to evaluate contagion processes within the parental couple. | At all time points, parental depressive scores were positively correlated and partner-related attachment showed small to moderate correlations with depressive symptom scores. Depressive symptoms at earlier time points significantly predicted depressive symptoms at later stages throughout the entire perinatal period. | These findings underscore the importance of including the partner of the affected parent when working to prevent and treat depressive states in the perinatal period. Thus, the finding provide indications of depressive symptom contagion in the perinatal period, in terms of a transmission of negative mood states from mothers to fathers around the time of birth. In accordance with our hypotheses, we found that partnerrelated attachment moderated the interparental relations of depressive symptoms. |
| 13 | Gastaldon et al., 2022 | Switzerland | Risk factors of postpartum depression and depressive symptoms: umbrella review of current evidence from systematic reviews and meta-analyses of observational studies | Depression | Systematic review and different review types (umbrela review) | Journal articles/reviews | A Measurement Tool to Assess Systematic Reviews (AMSTAR-2). | Including 185 observational studies (n = 3 272 093). | To assess the strength and credibility of evidence on risk factors of PPD, ranking them based on the umbrella review methodology. This umbrella review aimed to identify, quantify and measure the degree of credibility of the association of PPD with different risk factors, including per-ipheral markers, obstetric complications and psychological factors. | The 12 risk factors reported in the 11 systematic reviews were anaemia (during pregnancy and postpartum), GDM, Caesarean section, preterm delivery, intra-labour epidural analgesia, medically assisted conception, violent experiences, premenstrual syndrome (PMS), vitamin D deficiency and unintended pregnancy. | The most robust risk factors of PDD were premenstrual syn- drome, violent experiences and unintended pregnancy. These results should be integrated in clinical algorithms to assess the risk of PPD. |
| 14 | Hamimatunnisa et al., 2020 | Germany | Evaluation of antenatal risk factors for postpartum depression: a secondary cohort analysis of the cluster-randomised GeliS trial | Depression | Quantitative research | Journal articles/original research | Edinburgh Postnatal Depression Scale (EPDS), Pre-pregnancy BMI was self-reported. | Of the total 1583 participants, 6-8 weeks postpartum | we aimed to investigate associations between weight-related variables and PPD and to assess the influence of GWG on the risk for PPD. | Pre-pregnancy overweight or obesity were significantly positively associated with the odds of developing PPD, particularly among women with an antenatal history of anxiety or depressive symptoms. Participants who experienced PPD were more likely to have a lower educational level, be unmarried, smoke during early pregnancy, and suffer from antenatal anxiety and depressive symptoms. Being married significantly decreased the odds of PPD, whereas a low educational level was positively associated with the odds of PPD. | Pre-pregnancy overweight or obesity is associated with PPD independent of concurrent risk factors. History of anxiety or depressive symptoms suggests a stress-induced link between pre-pregnancy weight and PPD. |
| 15 | Harrisson et | United Kingdom | Supporting perinatal | Anxiety | Qualitative research | Journal articles/ori | a short, anonymous | 23 women who were | This study aimed to qualitatively explore | The themes were grouped around two broad domains that mapped onto the study's aims: | This work provides unique insight into potential sources of anxiety for women in the |

| | | | | | | | | | | | |
|---|---|---|---|---|---|---|---|---|---|---|---|
| | al., 2020 | | anxiety in the digital age; a qualitative exploration of stressors and support strategies | | | ginal research | screening survey which assessed their suitability in terms of the inclusion criteria. To identify women who had ex- perienced anxiety perinatally, we asked participants (1) how they rated their experience of anxiety from 1 to 5 (none, mild, moderate, severe, extremely severe); and (2) how much this distressed them on a scale of 1–5 (1 = not at all; 5 = severely). | either pregnant or within one-year postpartum. | women's experience of anxiety triggers and support in the perinatal period; and gain insight into what online support is acceptable for women with PNA. | un- derstanding women's sources of anxiety, and issues around support. Sources of anxiety: Unrealistic expectations of birth and motherhood (expectations of childbirth, expectations of breastfeeding, unrealistic guidlines and norms, unrealistic social comparison, societal pressure), imprtance of peer support (offline social support, online peer support, normalisation of experience), uncertanty and maternal confidence (unprepared for change, maternal confidence and overwhelm, conflicting or extreme, information). Issues around support: Stigma (internal and external stigma), lack of mental health support and knowledge (lack of postnatal healthcare support, mental health literacy). | perinatal period, while also offering potential internet-based support solutions that are likely to be acceptable and helpful for women with PNA. |
| 16 | Holopainen & Hakulinen, 2019 | Finland | New parents' experiences of postpartum depression: a systematic review of qualitative evidence | Depression | systematic review of qualitative evidence | Journal articles/reviews | he Edinburgh Postnatal Depression Scale (EPDS). | Thirteen papers that considered mothers (n= 199) who experienced PPD within a one-year postpartum period. | The aim of this review was to bring to light the experiences that both mothers and fathers have with PPD within a one-year postpartum period. | Four synthesized findings were developed: i) Depressed mothers feel unable to control their own lives due to low resilience; ii) The ambivalent feelings depressed mothers experience towards their babies, partners and in-laws cause distress and suffering; iii) Depressed mothers experience anger and despair if they perceive imbalances between their support needs and the support they get from healthcare providers and significant others; and iv) Depressed mothers experience hopelessness and helplessness resulting from their new-found motherhood and financial worries. | The qualitative studies concerning new parents' experiences of PPD have focused on the mother's perspective, and studies of the father's perspective, especially of the father's own experiences of PPD, are scarce. Both mothers and fathers do not receive enough support from their significant others. In addition, mothers want more support from health professionals. Because PPD has a great influence on the well-being of mothers and fathers, as well as children, it is important to understand what parents undergo after childbirth. |
| 17 | Kazmierczak et al., 2020 | Poland | Multivariate analysis of risk factors for postpartum depression | Depression | Quantitative research | Journal articles/original research | The research tools were a questionnaire of own design and the Edinburgh Postnatal | 70 women in the 4th week after child- birth, on average (min. 3 | The aim of the study is to assess the severity of postpartum depression and conduct multivariate analysis of risk factors. | Multi-dimensional logistic regression showed that age, anxiety due to motherhood and the occurrence of difficulties in looking after a child were the factors significantly associated with the symptoms of postpartum depression. This means that as the age of | The incidence of postpartum depression in the study sample did not differ significantly from the incidence reported in the literature. Worldwide. The risk of postpartum depression symptoms increased with age. Anxiety due to motherhood and difficulties in looking after a |

Appendix 4. Data extraction framework and variables charted from the included studies in the scoping review of psychosocial risk factors for postpartum depression and postpartum anxiety among European postpartum women

| | | | | | | | | | | | |
|---|---|---|---|---|---|---|---|---|---|---|---|
| | | | | | | | Depression Scale (EPDS). | weeks, max. 6 weeks after delivery), responded to the invitation. | | respondents increases, the level of postpartum depression also increases. It was confirmed that the women who felt anxiety due to motherhood presented significantly higher levels of postpartum depression disorders than the mothers who did not experience this condition. | child proved to be crucial determinants of depressive symptoms. Fertility at the level of three or more deliveries and individual personality types of women did not influence the occurrence of postpartum depression. |
| 18 | Kivirusu et al., 2020 | Finland | Trajectories of mothers' and fathers' depressive symptoms from pregnancy to 24 months postpartum | Depression | Quantitative research | Journal articles/original research | the 10-item version of the Center for Epidemiological Studies Depression Scale (CES-D), the STAI trait anxiety scale, a measure of family atmosphere ((e.g., approving (=1) – disapproving (=7); safe (=1) – unsafe (=7); quarrelsome (=1) – harmonious (=7)), the Perceived Stress Scale. | 1670 mothers (3, 8, and 24 months postpartum) | This study investigated trajectories of mothers' and fathers' depressive symptoms from prenatal to 24 months postpartum. Prenatal correlates of the trajectories were also examined. | Insomnia, earlier depression, anxiousness, stressfulness, and poor family atmosphere predicted the moderate and high (compared to low) depressive symptom trajectories among both mothers and fathers in multivariate analyses. Mother's higher depressive symptom trajectory was significantly associated with father's higher symptom trajectory. | Maternal and paternal depressive symptom trajectories from prenatal period up to two years postpartum seem stable, indicating the chronic nature of perinatal depressive symptoms. Mothers' and fathers' trajectories are associated with each other and their strongest predictors are common to both. |
| 19 | Klein et al., 2023 | Poland | Risk and protective factors for postpartum depression among Polish women – a prospective study | Depression | Quantitative research | Journal articles/original research | Edinburgh Postpartum Depression Scale (EPDS). immune Power Personality Questionnaire, Walsh Family Resilience Questionnaire. | 311 women in the postpartum period (4 weeks postpartum) | The study aims to identify risk and protective factors for postpartum depression (PPD) in Polish women and to assess the impact of pregnancy, delivery, the postpartum period, and psychosocial factors on PPD. | At 4weeks postpartum, the risk factors for PPD included: depressive episodes and the use of antidepressants before pregnancy, family history of depression, risk of preterm delivery, and feeling anxious about the newborn's health. Moreover, a mother's risk of developing PPD was associated with undertaking regular physical activity, concerns about the child's health, breastfeeding problems, using antidepressants, sleep problems, and sleeping 5h or less per day. | The results of our study demonstrate that PPD requires a multifactorial approach. Particular attention should be paid to screen- ing women postpartum as part of preventive health care programs to identify the risk factors for PPD. it will allow for further assessment of the severity of depressive symp- toms and implementation of appropriate treatment. |
| 20 | Lelievre et al., 2021 | Belgium | Postnatal depression: identification of risk factors in the short- | Depression | Quantitative research | Journal articles/original research | Edinburgh Postnatal Depression Scale (EPDS). | 131 women within 3 months of delivery. | In order to develop an efficient programme to trace vulnerable women after childbirth and to provide support within | In this study, two risk factors were identified for PND: negative feelings during pregnancy and the provision of only material support by the partner. | Women who experienced negative feelings during their pregnancy or women who experienced only material support from their partner were particularly at risk of presenting with PND within 3 months after delivery. |

| | | | | | | | | | | | |
|---|---|---|---|---|---|---|---|---|---|---|---|
| | | | stay maternity program in Belgium. A cross-sectional study | | | | | | primary care, the aim was to create an inventory of the risk factors for PND within the population of women participating in the short-stay programme. | | |
| 21 | Luciano et al., 2022 | Italy | Does antenatal depression predict post-partum depression and obstetric complications? Results from a longitudinal, long-term, real-world study | Depression | Quantitative research | Journal articles/original research | the Edinburgh Postnatal Depression Scale (EPDS) and an ad-hoc questionnaire on the women's sociodemographic, gynecological and peripartum characteristics as well as their psychiatric history. | A total of 268 pregnant women. Three time frames: 3 days, 1 months, 6 months after delivery. | Main aims of the present paper are to: (1) assess the prevalence of antenatal depression (AD) and identify its predictors; (2) analyse the impact of AD on obstetric outcomes and on the incidence of post-partum depression. | Predictors of AD were personal history of depression, a family history for depressive disorders and problematic relationships with the partner. Antenatal depression was a risk factor for higher EPDS scores at follow-ups. Other risk factors include the presence of a depressive disorder requiring a psychiatric treatment before pregnancy, family conflicts and financial difficulties. | Our results support the idea that women should be screened during pregnancy and post-partum for the presence of depressive and anxiety symptoms. Health professionals should be adequately trained to detect psychiatric symptoms during pregnancy. |
| 22 | Jimenez-Barragan et al., 2024 | Spain | Prevalence of anxiety and depression and their associated risk factors throughout pregnancy and postpartum: a prospective cross-sectional descriptive multicentred study | Anxiety and depression | Quantitative research | Journal articles/original research | Generalised Anxiety Disorder Scale (GAD-2), Edinburgh Postnatal Depression Scale (EPDS). | 335 woment at week 4-6 postpartum. | To assess the prevalence of anxiety and depression and their associated risk factors throughout the pregnancy and postpartum process using a new screening for the early detection of mental health problems. | The most relevant factors associated with positive screening for antenatal depression or anxiety during pregnancy, that appear after the first trimester of pregnancy, are systematically repeated throughout the pregnancy, and are maintained in the postpartum period were: a history of previous depression, previous anxiety, abuse, and marital problems. In weeks 12–14 early risk factors for positive depression and anxiety screening and positive EPDS were: age, smoking, educational level, employment status, previous psychological/psychiatric history and treatment, suicide in the family environment, voluntary termination of pregnancy and current planned pregnancy, living with a partner and partner's income. In weeks 29–30 risk factors were: being a skilled worker, a history of previous depression or anxiety, and marital problems. In weeks 4–6 postpartum, risk factors were: age, a history of previous depression or anxiety or psychological/psychiatric treatment, type of | Although the existence of different antenatal, perinatal, and postnatal depression and anxiety risk factors in this cohort is of concern, screening in the first trimester of pregnancy may improve the mental health status of preg-nant women, as well as reduce the subsequent burden in the mental health system. Care for young mothers in the early weeks and for older mothers during the postpartum period seems to be crucial. |

Appendix 4. Data extraction framework and variables charted from the included studies in the scoping review of psychosocial risk factors for postpartum depression and postpartum anxiety among European postpartum women

| | | | | | | | | | | | |
|---|---|---|---|---|---|---|---|---|---|---|---|
| | | | | | | | | | | treatment, having been mistreated, and marital problems. | |
| 23 | Molgora et al., 2022 | Italy | Predictors of Postpartum Depression among Italian Women: A Longitudinal Study | Depression | Quantitative research | Journal articles/original research | Wijma Delivery Experience Questionnaire; State-Trait Anxiety Inventory; Edinburgh Postnatal Depression Scale; Parenting Stress Index (Short Form); Dyadic Adjustment Scale; and Multidimensional Scale of Perceived Social Support. | The 137 women up to 12 months postpartum | The present study aimed to describe the psychological status of mothers up to 12 months postpartum, and to investigate the predictors of depressive symptoms at 12 months postpartum, considering obstetric factors along with psychological and relational variables. | The quality of childbirth experience and trait anxiety at three months postpartum emerged as significant predictors of postpartum depression at 12 months. Women who had experienced one or more stressful events (e.g., economic problems, work problems, own illness, or illness of a significant person, etc.) during pregnancy or in the postpartum reported greater depressive symptoms at 12 months postpartum. Finally, the quality of childbirth experience at three months had a significant impact on PPD, with women reporting a critical or even traumatic experience showing higher levels of depressive symptoms at 12 months postpartum. The findings of this analysis showed statistically significant results for two predictors: the quality of childbirth experience and trait anxiety. | Our findings highlight the importance of providing stable programs (such as educational programs) to mothers in the first year postpartum. Furthermore, because the quality of the childbirth experience is one of the most important predictors of PPD at 12 months postpartum, effort should be made by healthcare professionals to guarantee a positive experience to all women to reduce possible negative long-term consequences of this experience. Regarding the main predictors of PPD at 12 months postpartum (the second aim of our study), our findings showed significant associations with the quality of childbirth experience and trait anxiety |
| 24 | Smorti et al., 2019 | Italy | A Comprehensive Analysis of Post-partum Depression Risk Factors: The Role of Socio-Demographic, Individual, Relational, and Delivery Characteristics | Depression | Quantitative research | Journal articles/original research | the Beck Depression Inventory (BDI), the State Anxiety Inventory (STAI_Y2). | 161 women, 1 months after delivery | (1) to confirm previous results exploring the role that several sets of variables, such as socio-demographic, individual, relational, and related to delivery characteristics, separately considered, play as risk factors for the onset of postpartum depression; and (2) to verify which, among the above risk factors, have a more significant influence when they are considered together. | A high level of PPD was associated with a high level of prenatal anxiety and depression. Moreover, prenatal anxiety and depression were significantly and positively correlated. Overall prediction of the severity of PPD, which are: age, anxiety, depression, parental relationship, romantic relationship, prenatal attachment, typology of delivery, and epidural analgesia. Overall, the level of prenatal attachment to child was the most important predictor of post-partum depression. | These findings emphasize the very important role of prenatal attachment for the onset of postpartum depression and the need to promote adequate and targeted prevention interventions. |
| 25 | Sunnqvist et al., 2019 | Sweden | Depressive symptoms during pregnancy and postpartum in women and use of antidepressant treatment – | Depression | Quantitative research | Journal articles/original research | The Edinburgh Postnatal Depression Scale (EPDS), the NorVold Abuse Questionnaire, and a questionnaire about | 731 from 1 to 1.5 years postpartum | The aim of this study was to investigate whether women, who reported "symptoms of depression" during pregnancy and up to 1.5 years postpartum, who reported domestic violence or not, were treated with | Women with scores of depressive symptoms were significantly younger, more often speaking a foreign language at home, single/living apart, less educated, unemployed, financially distressed, and smokers. We also found that women with symptoms of depression, besides being socially vulnerable, also had a risk factor in having been exposed to domestic violence. | This study shows the importance of detecting depressive symptoms during early pregnancy and a need for standardized screening methods. The findings show the clinical challenge in detecting this important group of women during an especially vulnerable time in their life. The need for standardized screening methods for depression both during pregnancy and postpartum is emphasized. |

| | | | | | | | | | | | |
|---|---|---|---|---|---|---|---|---|---|---|---|
| | | | a longitudinal cohort study | | | | medication during pregnancy. | | antidepressant medication. | | |
| 26 | Urbanova et al., 2021 | Slovakia | The Association between Birth Satisfaction and the Risk of Postpartum Depression | Depression | Quantitative research | Journal articles/original research | A 30-item version of the Birth Satisfaction Scale (BSS) and the Edinburgh Postnatal Depression Scale (EPDS), the Perceived Stress Scale (PSS). | The study included 584 women 2 to 4 days postpartum. | The aim of the present study was to explore the relationship between childbirth satisfaction and the level of postpartum depression, considering other factors that could affect the risk of postpartum depression. | A lower level of satisfaction with childbirth was a significant predictor of a higher risk of PPD. Levels of prenatal stress, psychiatric history, parity and type of delivery were also significantly associated with the levels of postnatal depression. Other risk factors that increased the risk of PPD were: stress (moderate stress), type of childbirth (caesarean delivery), parity (primiparity) and a positive psychiatric history. The same risk factors that were associated with the risk of developing PPD also negatively affected birth satisfaction. | The current study confirmed the association between the level of birth satisfaction and the risk of developing PPD, i.e., a lower satisfaction with childbirth may increase the risk of developing PPD. |
| 27 | Zee-van-den-berg et al., 2021 | Netherlands | Postpartum depression and anxiety: a community-based study on risk factors before, during and after pregnancy | Anxiety and depression | Quantitative research | Journal articles/original research | the Edinburgh Postnatal Depression Scale (EPDS) and 6-item State-Trait Anxiety Inventory (STAI-6). | We used data from 1406 mothers. Risk factors were collected at 3 weeks and 12 months postpartum. | Our aim was to identify risk factors for postpartum depression and anxiety, before, during and after pregnancy. | Factors associated with higher risk of depression were: foreign language spoken at home, history of depression, low maternal self-efficacy and poor current health of the mother. We found four factors from the postpartum period associated with a higher risk of depression; two were related to breastfeeding and the other two to maternal self-efficacy and current health of the mother. Early cessation of breastfeeding, breastfeeding self-efficacy (women who feel unsure about their ability to breastfeed successfully), low self-efficacy (a mother's confidence in her ability to care for her child), poor maternal health condition. Factors associated with higher risk of anxiety were: higher educational level, history of depression, preterm birth, negative experience of delivery and first week postpartum, excessive infant crying, low maternal self-efficacy, low partner support and poor current maternal health. We found five factors from the postpartum period to be associated with anxiety: low support by the partner, a negative experience of the first week postpartum, experiencing the infant's crying as excessive, poor current health of the mother, and low maternal self-efficacy, the latter with a very high odds ratio. | This study has found both shared and separate risk factors for post- partum depression and anxiety, and suggested the importance of the timing of these factors before, during, and after pregnancy. Our findings thus provide valuable information for development of preventive in-terventions and treatment to improve the mental well-being of mothers during the postpartum period. |
| 28 | Zejnullahu et | Albania | Prevalence of postpartum | Depression | Quantitative research | Journal articles/ori | the Edinburg Postnatal | A total of 247 | The aim of this study was to examine the | Four predictor variables for postpartum depression: pregnancy complications; fear of | Current findings highlight importance of early identification of high-risk women for |

| | | | | | | | | | | | |
|---|---|---|---|---|---|---|---|---|---|---|---|
| | al., 2020 | | depression at the clinic for obstetrics and gynecology in Kosovo teaching hospital: Demographic, obstetric and psychosocial risk factors | | | ginal research | Depression Scale (EPDS). | delivery women were screened for postpartum depression at 6 weeks postpartum | prevalence and risk factors of postpartum depression at the Clinic for Obstetrics and Gynecology in Pristina, Kosovo (a tertiary referral center). | childbirth; prenatal depression or anxiety; poor marital relation. We found a statistically significant association between the perinatal complications and postpartum depression. Among psychological factors fear of childbirth and depression or anxiety during this pregnancy was significantly associated with postpartum depression . Poor marital relation was associated with postpartum depression. Our data confirmed that perinatal complications are associated with increased risk for postpartum depression. | postpartum depression and necessary interventions during the antenatal care. Psychoeducation and psychotherapy are mandatory through pregnancy and after delivery, especially in a group of women with psychological morbidity. The high prevalence of postpartum depression in our setting and its known adverse effects on woman, infant and her family implies an urgent need for evidence-based interventions. Such interventions are needed to promote knowledge of perinatal mental illness and improve maternal mental health in particular in less developed countries. |
| 29 | Zikic et al., 2024 | Serbia | Depression in the Perinatal Period: Course and Outcome of Depression in the Period from the Last Trimester of Pregnancy to One Year after Delivery in Primiparous Mothers | Depression | Quantitative research | Journal articles/original research | the Edinburgh Postnatal Depression Scale, Toronto Alexithymia Scale, Beck Anxiety Inventory, and The Mood Disorder Questionnaire. | 188 women at the first, sixth, and twelve months postpartum. | Our study aims to determine the frequency and course of depressive symptomatology in the perinatal period with particular reference to objective rate and outcome of postpartum depression. | Our results suggest that the most important risk factor for new-onset postpartum depression was alexithymia in the third trimester and dissatisfaction with socioeconomic status. In our study, a significant risk factor for new-onset depression was suicidality in the family and the absence of an emotional partner. Social support and dissatisfaction in marriage or with the partner are some of the most common social risk factors, along with stress and current or past abuse. Being single with an unsatisfactory economic status, suicidality in the family, anxiety, alexithymia and absence of complications during childbirth were associated with new-onset, postnatal depression. | Our results imply that a new onset of depression is most intensive during the first six months, and after that, it is sporadic. Further studies are needed to explore whether all depressive symptomatology in the postnatal period is the same, or perhaps postpartum depression, classified in this way, has specific characteristics, etiology, and consequently different treatment and preventive options. |
| 30 | Zyrek et al., 2024 | Poland | Social support during pregnancy and the risk of postpartum depression in Polish women: | Depression | Quantitative research | Journal articles/Scientific reports | Berlin Social Support Scales, the Edinburgh Postpartum Depression Scale (EPDS). | 932 women at four weeks after labor | our study aimed to examine the association between the risk of postpartum depression (PPD) and perceived social support during pregnancy. | Higher perceived available support (emotional and instrumental), currently received support (emotional, instrumental and informational), satisfaction with the support, and sum of score were all associated with lower risk of PPD, after controlling for selected covariates (woman's age, socioeconomic status, parity status, place of residency, education, child's Apgar score, type of delivery, complications during birth, kin assisting the labor, breastfeeding). | Our results suggest that the more social support the pregnant woman receives, the lower is her risk of PPD. Since humans evolved as cooperative breeders, they are inherently reliant on social support to raise children and such allomaternal help could improve maternal well-being. |

Appendix 5. Characteristics of studies included in the scoping review of psychosocial risk factors for postpartum depression and postpartum anxiety among European postpartum women

Characteristics of included studies

| Analysed information unit | Data overview | N (30) |
|---|---|---|
| Publication years | 2019<br>2020<br>2021<br>2022<br>2023<br>2024 | 5<br>5<br>4<br>7<br>3<br>6 |
| Countries | Albania<br>Belgium<br>Netherlands<br>Serbia<br>Slovakia<br>Switzerland<br>United Kingdom<br>Germany<br>Finland<br>France<br>Portugal<br>Norway<br>Poland<br>Spain<br>Sweden<br>Italy | 1<br>1<br>1<br>1<br>1<br>1<br>1<br>1<br>2<br>2<br>2<br>2<br>3<br>3<br>3<br>5 |
| Types of articles | Journal articles: original research/report<br>Journal articles: reviews | 28<br>2 |
| Design | Longitudinal/cohort study design<br>Cross-sectional design<br>Qualitative study design<br>Systematic reviews and other types of reviews | 20<br>7<br>1<br>2 |
| Aim of studies | To identify risk factors<br>To explore the relationship/ association<br>To explore experience | 19<br>9<br>2 |
| The type of disorder examined in the study | Depression<br>Anxiety<br>Depression and Anxiety | 21<br>3<br>6 |

© 2026 Zelčāne E. et al.
